# Designing a parallel evolutionary algorithm for inferring gene networks on the cloud computing environment

**DOI:** 10.1186/1752-0509-8-5

**Published:** 2014-01-16

**Authors:** Wei-Po Lee, Yu-Ting Hsiao, Wei-Che Hwang

**Affiliations:** 1 Department of Information Management, National Sun Yat-sen University, Kaohsiung, Taiwan

**Keywords:** Gene network inference, Systems biology, Evolutionary algorithm, Swarm intelligence, Parallel model, Cloud computing, MapReduce

## Abstract

**Background:**

To improve the tedious task of reconstructing gene networks through testing experimentally the possible interactions between genes, it becomes a trend to adopt the automated reverse engineering procedure instead. Some evolutionary algorithms have been suggested for deriving network parameters. However, to infer large networks by the evolutionary algorithm, it is necessary to address two important issues: premature convergence and high computational cost. To tackle the former problem and to enhance the performance of traditional evolutionary algorithms, it is advisable to use parallel model evolutionary algorithms. To overcome the latter and to speed up the computation, it is advocated to adopt the mechanism of cloud computing as a promising solution: most popular is the method of MapReduce programming model, a fault-tolerant framework to implement parallel algorithms for inferring large gene networks.

**Results:**

This work presents a practical framework to infer large gene networks, by developing and parallelizing a hybrid GA-PSO optimization method. Our parallel method is extended to work with the Hadoop MapReduce programming model and is executed in different cloud computing environments. To evaluate the proposed approach, we use a well-known open-source software GeneNetWeaver to create several yeast *S. cerevisiae* sub-networks and use them to produce gene profiles. Experiments have been conducted and the results have been analyzed. They show that our parallel approach can be successfully used to infer networks with desired behaviors and the computation time can be largely reduced.

**Conclusions:**

Parallel population-based algorithms can effectively determine network parameters and they perform better than the widely-used sequential algorithms in gene network inference. These parallel algorithms can be distributed to the cloud computing environment to speed up the computation. By coupling the parallel model population-based optimization method and the parallel computational framework, high quality solutions can be obtained within relatively short time. This integrated approach is a promising way for inferring large networks.

## Background

### Model

Gene regulatory networks (GRNs) play important roles in genetic systems and are involved in various biological processes during the development of living organisms. Through analyzing the interactions between genes, we can uncover some complex behavior patterns and study genetic systems in detail. Gene network construction has been considered to be one of the most important issues in systems biology research. It is a procedure used to manipulate experimentally measured time-series data for building a model that can describe the observed phenotypic behavior of a system to be studied. To save the effort of testing experimentally which interactions in the gene networks are possible and then deriving the network accordingly, an automated reverse engineering procedure has been advocated [1,2]. This work means to establish a practical computational methodology for the inference of real gene networks.

To infer a network with the desired system behavior, the crucial steps are to select a network model and then to find the most suitable structural parameters for the network. Many models have been proposed to address different levels of biological details, ranging from the very abstract (involving Boolean values only) to the very concrete (including full biochemical interactions with stochastic kinetics). To capture the underlying physical phenomena of a gene network, this work adopts one of the most popular concrete models, the S-system model, to represent a gene network. The S-system model is a type of ordinary differential equation (ODE) model. It comprises a specific set of tightly coupled ODEs in which the component processes are characterized by power law functions [2,3]. In the coupled S-system model, the systematic structure can be described as:

(1) $$\frac{dx_{i}}{\text{dt}}=\alpha_{i}\overset{N}{\underset{j=1}{\prod }}x_{j}^{g_{i,j}}-\beta_{i}\overset{N}{\underset{j=1}{\prod }}x_{j}^{h_{i,j}}$$

In the above equation, *x*_*i*_ is the expression level of gene *i* and *N* is the number of genes in a genetic network. The non-negative parameters *α*_*i*_ and *β*_*i*_ are rate constants that indicate the direction of mass flow. The real number exponents *g*_*i,j*_ and *h*_*i,j*_ are kinetic orders that reflect the strength of the interactions from gene *j* to *i*. The above set of parameters defines an S-system model. To infer a coupled S-system model is, therefore, to determine all of the 2*N*(*N* + 1) parameters simultaneously.

The above inference of an S-system model is a large-scale parameter optimization problem that is very time-consuming. After analyzing the structural characteristics of gene networks, Maki *et al*. proposed an efficient strategy to divide this inference problem into *N* separated small sub-problems, each of which corresponds to one gene [4]. In other words, in a decoupled S-system, the original tightly coupled system of non-linear differential equations is decomposed into several differential equations [5,6], each of which describes a specific gene that can be separately inferred. Although addressing gene network inference in the above-described way can substantially reduce the computational complexity, it is notable that the computational cost will still grow linearly when inferring large networks.

One major goal in gene network reconstruction is to minimize the accumulated discrepancy between the gene expression data recorded in the data set (the desired values) and the values produced by the inferred model (the actual values). The performance of a certain model can be defined directly as the mean squared error (MSE) over the time period. For the decoupled model described above, the evaluation function of the *i*-th sub-problem can thus be given as the following:

(2) $$\text{MSE}\left(i\right)=\overset{T}{\underset{t=1}{\sum }}{\left\{\frac{x_{i}^{a}\left(t\right)-x_{i}^{d}\left(t\right)}{x_{i}^{d}\left(t\right)}\right\}}^{2},\text{for}\;i=1,2,\ldots ,N$$

where $x_{i}^{d}\left(t\right)$ is the desired expression level of gene *i* at time *t*,  $x_{i}^{a}\;\left(t\right)$ is the value generated from the inferred model, *T* is the number of time points in measuring gene profiles, and *N* is the number of genes in the network.

In real-world situations, the number of data points available is often smaller than that of the parameters to be determined. It is thus possible to obtain many feasible solutions with various combinations of network parameters (meaning different network structures/topologies). To solve the structure problem, prior knowledge or assumptions are required. Some researchers proposed to incorporate the structural/topological properties of the biological networks (such as the degree distribution of the nodes in a scale-free network or the presence of network motifs) with the gene expression data in the evaluation process. For example, some inference methods intend to limit the number of GRN connections to be as small as possible because gene regulatory networks are typically known to be sparsely connected. In such a case, a small penalty term that measures the connection between the genes can be added to the fitness function to discourage the connections [7,8]. There are also other researchers who suggest using expert knowledge to determine the models, such as taking the form of parameter constraints to account for the prior domain knowledge to restrict the search of network parameters [9].

It is worth noting that prior knowledge is not always available. Additionally, the topological properties within the relatively small size genome-scale networks are not sufficient enough to provide practical information, even if they have been studied in sophisticated network modeling to better understand the corresponding biological details. Therefore, without losing generality, in this work, we simply take the mean square error (as shown in equation ( [2])) to define evaluation function. We focus on how to design a parallel computational model to infer decoupled S-system and how to distribute the relevant computation to a cloud computing environment.

### Algorithm

Although the non-linear ODEs mentioned above can more accurately capture the system dynamics of gene networks, they are difficult to solve by traditional local optimization techniques [3,10], such as the conjugate gradient method, Newton’s method, and the Nelder-Mead simplex method. These techniques rely on the derivative of the evaluation function or a comparison of the evaluation function at the vertices of a simplex in the parameter space. Thus, they are not suitable for the optimization problem here. Global optimization techniques for parameter estimation are better choices than the above-described local techniques for finding the global optimum and are more suitable for biological systems.

Among global parameter estimation methods, the methods that use deterministic strategies are superior for finding the global optimum, but these are computationally more expensive. In contrast, the methods with stochastic strategies can have solutions that are near the global optimum within a reasonable amount of time. Population-based approaches (such as evolutionary algorithms, EAs) are stochastic methods, and they have been used in many studies to infer the S-system model (e.g., [5,6,11-13]).

Adopting a reverse-engineering approach to derive GRNs in their early work, Tominaga and his colleagues employed a conventional simple genetic algorithm (SGA) to infer genetic networks with the S-system model [14]. Their results showed that SGA can successfully evolve small scale GRNs (around 5 genes). Following Tominaga’s work, Kikuchi *et al.* proposed an incremental optimization strategy with a novel crossover technique [5] for network inference. The modified SGA has a better performance while inferring the S-system model; however, the modified approach is computationally expensive since it has to deal with too many parameters simultaneously. Ho *et al.* also proposed an intelligent two-stage evolutionary algorithm (iTEA) that used orthogonal matrices to decide better solutions [7]. iTEA has shown its great power in the reconstruction of gene networks, but the orthogonal matrices will become bigger for large scale networks (e.g., 30 genes) and the computational time thus increases dramatically. There are other works proposed for further performance improvement, for example, [5,13,15-18]. However, they share the common problem of scalability. To tackle this problem, we adopt this type of global optimization technique and develop a new parallel model to enhance the search performance and speed up the computational process.

Using EAs to infer large gene networks that involve a large number of genes, two inherent features of EAs must be considered seriously. The first is premature convergence, which has the negative effect of losing population diversity before the goal is reached. To overcome this problem, parallel-model EAs have been proposed to divide the original population into several subpopulations to maintain population diversity, and their efficiency has been confirmed (e.g., [19,20]). The other problem that must be addressed is the inordinate amount of time that is required to perform evaluations for all of the individuals. To enhance the search performance and speed up the computation at the same time, it is therefore critical to develop parallel models for EAs and execute the parallel models in high-performance computing environments.

### Computational platform

As mentioned above, a decoupled S-system model with *N* genes decomposes the original non-linear differential of S-system into *N* differential equations. It means that the original task that contains 2*N*(*N* + 1) parameters altogether is now transferred into *N* sub-tasks; each sub-task has 2(*N* + 1) parameters (i.e., *α*_*i*_, *g*_*i,*1..*N*_, *β*_*i*_, *h*_*i,*1..*N*_ for a specific gene *i*) and can be independently performed. The decoupled S-system model allows us to perform the sub-tasks in a parallel way and then to assemble the results to obtain a final complete model. This characteristic perfectly matches the pre-requirement of distributed computing. Moreover, the population-based inference algorithm can also be parallelized to speed up the computation. Combining the above ways of parallelism, large scale networks containing hundreds or thousands of genes can thus be inferred.

Conceptually, the central idea of the parallel EA is to divide a large population used in a sequential version of EA into multiple smaller subpopulations and to distribute them on separate computational nodes so as to perform the evaluations on separate processors concurrently. Although parallel computers can be used to speed up EAs, they are expensive and usually not available in a campus environment. One promising choice is to utilize cloud computing, and the MapReduce programming model provides an easy-to-implement framework with fault tolerance capabilities [21-23]. This framework has been used to successfully solve many large-scale scientific computing problems, including problems in the life sciences [24-26].

**Figure 1:**
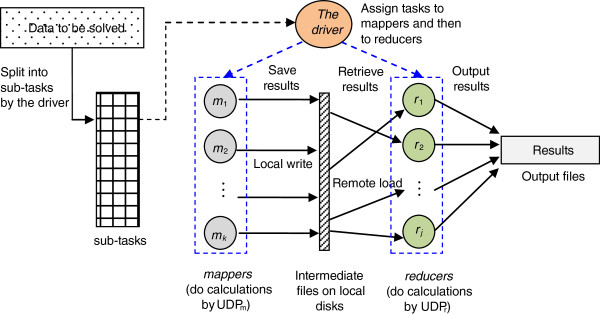
The general workflow of a MapReduce process.

The goal of MapReduce is to deploy a large amount of time- and memory-consuming tasks to all computing nodes that process tasks in parallel by user-defined algorithms. Figure  1 shows the overall flow of a MapReduce process in which one master and many slave machines are organized. The MapReduce framework contains two main phases, *map* and *reduce*, that are controlled by the master machine (i.e., by the *driver* program in it). In the map phase, the driver loads the input data, divides it into sub-tasks for the computing nodes (they are slave nodes and named mappers in this phase), and instructs these nodes to perform some calculations according to the user-defined program for mappers (UDP_m_). The results are saved to immediate files. In the reduce phase (after the calculations on mappers have been completed), the driver asks the computing nodes (namely, reducers in this phase) to collect the results from the intermediate files and requests them to execute the user-defined program for reducers (UDP_r_). Then reducers combine all the sub-results to form the output. If the calculations must be performed iteratively, the driver will continuously repeat the above two phases until the stopping criterion is met. Parallelization in the MapReduce framework is achieved by executing multiple Map and Reduce tasks concurrently on different machines in the cluster that runs the model. This framework deals with almost all of the low-level details, including the data distribution, communication, fault tolerance, etc. In this way, the users can concentrate on the algorithms and define the map/reduce methods for their applications. An example is given about how to use the above MapReduce method to perform computation for a decoupled S-system in the Additional file 1.

Apache Hadoop is an open source implementation of MapReduce written in Java. Apart from MapReduce, Hadoop also provides the Hadoop Distributed File System (HDFS) to reliably store data across hundreds of computers. The cluster is a single master cluster with a varying number of slave nodes. The slave nodes can act as both the computing nodes for MapReduce and as data nodes for the HDFS. Hadoop MapReduce has been used in several bioinformatics research studies [27,28]. For example, it has been employed to develop algorithms for the analysis of next-generation sequencing data [29,30], to implement systems for sequence alignment [31], and to develop proteomic search engines [32].

Although the Hadoop MapReduce framework can effectively reduce the runtime for large-scale data-intensive applications, it has some limitations [33]. One limitation is job latency, which means the time spent on running background tasks, such as the time spent to schedule, start, and finish a MapReduce job. The other limitation is input data processing, which means the need to read the input data from the HDFS every time a MapReduce job is executed. These limitations decrease the efficiency of the framework, especially in applications that involve iterative computations. An alternative MapReduce framework is Twister, which can store input data in memory between iterations [34]. However, storing data in this way has another disadvantage: it requires the data to fit in the collective memory of the cluster so that the framework can be effective; this approach is unfeasible for tasks that have a large amount of data. In addition, Twister has a weak fault tolerance capability, but this capability is very important for population-based computations that require a large number of iterations and must be protected from hardware or network failures.

To infer large gene networks from the expression profiles, in this work, we present a parallel evolution-based optimization algorithm and distribute the algorithm to the cloud computing environment to speed up the computation. Considering the critical factors and the current use popularity, we chose to use Hadoop MapReduce for our application. To evaluate the proposed approach, experiments have been conducted, and the results have been analyzed. They show that our approach can be successfully used to infer networks with desired behaviors from four real-world biological datasets. Also, the evolution algorithm with a parallel model can improve the performance of network inference. Most importantly, using the MapReduce framework, the computation time for the inference algorithm can be substantially reduced, such that large networks can be inferred.

## Methods

### Optimization algorithm

**Figure 2:**
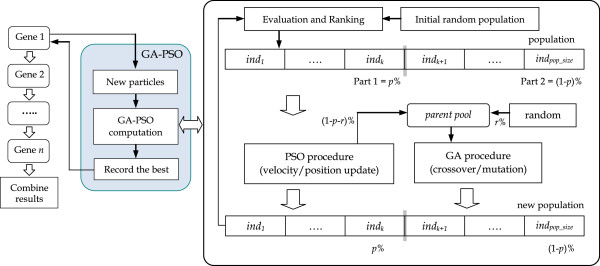
The main flow of our optimization algorithm.

To infer network parameters, we adopt a hybrid population-based approach that includes both genetic algorithm (GA) and particle swarm optimization (PSO) procedures to exploit their respective advantages. This approach is revised from an algorithm that we developed previously for parameter optimization while evolving a fully connected gene network as a whole. The original version of our optimization algorithm has been shown empirically to outperform other relevant algorithms on many popular benchmark problems [16], because it can effectively achieve a balance between local search (exploitation) and global search (exploration). In this study, we make some modifications and apply the revised algorithm to the decomposed S-system problem. As described in the background section, the set of parameters that correspond to each gene in the decoupled S-system model can be inferred separately. Therefore, our hybrid algorithm is used to optimize the set of network parameters for each gene sequentially, as illustrated in Figure 2 (only the flow for Gene 1 is indicated as an example). In this figure, the *ind*_*i*_ (1 ≦ *i* ≦ *pop_size*) is a possible solution (i.e., named an individual in GA or a particle in PSO) included in the population for a specific gene. After the parameters for all of the genes are determined, they are combined to form the solution. The following subsections briefly describe how the proposed approach operates in both sequential and parallel ways.

#### The sequential model

The first step of using a population-based search method to solve an optimization problem is to define an appropriate representation to characterize the problem’s solution. To infer a gene network, we take a direct encoding scheme to represent solutions for both the GA and PSO parts, in which the network parameters related to the decoupled S-system model are arranged as a linear string chromosome of floating-point numbers. That is, for each gene *i* in a network with *N* gene nodes, the solution is represented as (*α*_*i*_, *g*_*i,*1_, *g*_*i,*2_, …, *g*_*i,N*_, *β*_*i*_, *h*_*i,1*_, *h*_*i,2*_, …, *h*_*i,N*_). As the parameter range of kinetic orders *g*_*i,*1*~N*_, *h*_*i,*1*~N*_ ∈[-3, 3] have been widely used as the default search space for evolutionary algorithms (e.g. [5,7,8,17]), we thus choose to use the same range in this study. For the rate constants, several range settings have been taken, such as *α*_*i*_, *β*_*i*_ ∈[0, 10], [0, 15], and [0, 20] in the literature (e.g. [5,8,17]). Based on a preliminary test, we found no significant difference for using these ranges. The rate constants represent the ratio between the synthesis and degradation process. For simplicity, the range from 0 to 10 is considered suitable to evaluate the constant rate between the two processes. Hence, in this study the ranges for *α*_*i*_, and *β*_*i*_ are both set to [0, 10].

The next step is to define a fitness function for the evaluation of candidates and to use the results as a form of selective pressure to derive better solutions. In this study, we directly use the mean squared error function shown in the above section (i.e., equation ( [2])) as the fitness function. With the above representation and evaluation function, we can then perform the hybrid approach to determine the network parameters.

As shown in Figure  2, in this algorithm a random population is first generated and evaluated. Then, the individuals (particles) are ranked according to their fitness and are separated into two parts. The first part includes elites (i.e., the best *p*% individuals of the entire population); these individuals are preserved and enhanced by the PSO procedure and are sent directly to a candidate list that is being prepared for the next generation. The second part includes individuals with a lower performance compared with those in the first part (i.e., the worst (1 - *p*)% individuals); they are discarded. To replace the removed individuals, the same number of individuals is produced to form a parent pool, in which some individuals are randomly generated (i.e., *r*%), and the remainder (i.e., (1 - *p* - *r*)%) are randomly selected from the ones that were already improved by the PSO procedure. Then, this parent pool is used to create new individuals through the GA procedure, and the newly created individuals are sent to the candidate list. Once a new candidate list is formed, the individuals in this list are again ranked according to their fitness values, and the new population is used for the next generation. The above procedure is repeated until the termination criterion is met.

In our experiments, *p* has a fixed value (which is estimated from a preliminary test), while the randomness rate *r* is a variable whose value can be changed during the run to control the population diversity (i.e., to coordinate the progress of the PSO and GA parts). For example, the randomness rate can increase linearly in proportion to the generation number to maintain the overall population diversity; the PSO tends to perform a local search at the end of the run, which implies that a high rate of randomness is desirable.

In the PSO procedure shown in Figure  2, the particles are potential solutions moving in the search space defined by the parameters. Each particle has its own position and velocity. The position is the current value of the model parameter, while the velocity is a vector of numbers that are added to the position coordinates of the particle in order to move the particle from one time step to another. To enhance the individual performance, the main operator here is velocity updating for the particles, which combines the best position reached by the swarm of particles and the best position reached by a certain particle during its movement history. The algorithm causes the particles to move toward the best position in the swarm. In this study, the velocity and the position of a particle at time step *t* + 1 are updated from those at the previous time step *t* by the following rules (which were modified from the original PSO [35]):

(3) $$v_{\text{id}}^{t+1}=\chi \left(wv_{\text{id}}^{t}+c_{1}r_{1}^{t}\left(p_{\text{id}}^{t}-x_{\text{id}}^{t}\right)+c_{2}r_{2}^{t}\left(p_{\text{gd}}^{t}-x_{\text{id}}^{t}\right)\right)$$

(4) $$x_{\text{id}}^{t+1}=x_{\text{id}}^{n}+v_{\text{id}}^{t+1}$$

(5) $$\chi =\frac{2}{\left|2-\varphi -\sqrt{\varphi^{2}-4\varphi }\right|}$$

In the above equations, *v*_*id*_ and *x*_*id*_ are the velocity and position of particle *i* in dimension *d*, *p*_*id*_ is the previous best position of particle *i*, *p*_*gd*_ is the best position in the swarm, and *w* is the inertia weight, which controls the momentum of the particle by weighting the contribution of the particle’s previous velocity. The coefficients *c*_1_ and *c*_2_ are two positive acceleration constants; they are often determined empirically. The variables *r*_1_ and *r*_2_ are random values within the range [0, 1]. The products *c*_1_*r*_1_ and *c*_2_*r*_2_ thus stochastically control the overall velocity of a particle. In addition, *χ* is the constriction factor that was originally introduced in [35] to constrict the velocities of particles and to achieve an exploration-exploitation balance during the search. Finally, *φ* is a parameter often used to control the convergence characteristics of the PSO. It is the summation of *c*_1_ and *c*_2_, and has to be larger than 4.

As mentioned above, the GA part is used for the creation of new individuals to replace the individuals who were discarded. In this procedure, the tournament selection strategy is employed to choose parent pairs. For the selected particles *old*_1_ and *old*_2_, the crossover operator is implemented as described below to create two new particles *new*_1_ and *new*_2_:

(6) $$\begin{array}{ll} \;{\text{new}}_{1}\left(x_{\text{id}}\right) & =\left({\text{old}}_{1}\left(x_{\text{id}}\right)+{\text{old}}_{2}\left(x_{\text{id}}\right)\right)/2-\varphi_{1}\\ & \;\times {\text{old}}_{2}\left(v_{\text{id}}\right) \end{array}$$

(7) $$\begin{array}{ll} \;{\text{new}}_{2}\left(x_{\text{id}}\right) & =\left({\text{old}}_{1}\left(x_{\text{id}}\right)+{\text{old}}_{2}\left(x_{\text{id}}\right)\right)/2-\varphi_{2}\\ & \;\times {\text{old}}_{1}\left(v_{\text{id}}\right) \end{array}$$

In this operator, *φ*_1_ and *φ*_2_ are uniform random variables (different from the parameter *φ* in equation (5)) that have values between 0 and 1. If the position of a newly created particle falls outside the specified range, it is set to the maximal value allowed (i.e., *x*_*id*-max_). As can be observed, this operator mainly serves to incorporate the velocities of the two parents; it produces two children with positions between the two parents but that accelerate away from the current location to increase the population diversity. Then, the non-uniform mutation is performed to fine-tune the numerical values of the individuals. This operator causes a parameter *p*_*k*_ in a selected individual to be changed to *p*_*k*_′, where

In the above equations, *LB* and *UB* are the lower and upper bounds of the parameter *p*_*k*_ and *t* is the iteration number. The function Δ(*t*, *y*) (the often-used function as described in [36]) returns a value between 0 and *y* such that the probability of Δ(*t*, *y*) will be close to 0 as *t* increases.

#### The parallel model

The GA-PSO approach described above has been extended with a parallel model to enhance the search performance and to reduce the computational time in the network inference. Conceptually, parallelizing the above procedure involves dividing a large population into multiple smaller subpopulations so that they can be addressed on separate computational nodes simultaneously. Depending on the subpopulation size, two types of parallel models are typically used: coarse-grained and fine-grained. The best choice depends mainly on the machine availability and the type of application task. Because of the hardware limitation, in this work we adopt a coarse-grained model and distribute the computation in the cluster computing environment to achieve parallelism.

A coarse-grained model divides the whole population into a small number of subpopulations; each subpopulation can be evaluated by the original GA-PSO independently on a separate processor. In this model, any change for a particle occurs only in a local subpopulation. A communication (migration) phase is introduced in the parallel model to periodically select some promising individuals from each subpopulation and send them to other subpopulations. A common strategy is to use the selected individuals to substitute for the worst individuals of the neighboring subpopulations. In this way, the algorithm has a higher probability of maintaining population diversity and protecting good solutions that are found locally.

Our coarse-grained GA-PSO performs island model parallelism (called iGA-PSO). Ideally, each subpopulation is distributed to one or more available computational node(s). In this way, the subpopulations can be independently executed at the same time to speed up the solution search process. However, because the MapReduce framework is adopted in this work to realize the distributed computing, the dispatch of islands to the computational nodes has to follow the corresponding design principles and to work in a non-traditional manner. For example, the particles in the same island may be arranged in different computational nodes in the map phase and later grouped together in the reduce phase. Here, we focus on the operational flow of iGA-PSO in the conceptual level. How the iGA-PSO works on the MapReduce framework is described in the following section.

The distributed iGA-PSO code continues for a certain number of iterations before migration occurs. Because of the variation in the machine specifications (e.g., the CPU and memory utilities) and the discrepancy of the evaluation time in the individuals, computers that run different subpopulations can take different amounts of time to complete the corresponding evolution. To perform migration as in the original island model, our work uses a synchronized strategy to organize the distributed computation. In other words, the communication phase can start only when all of the subpopulations have been executed for a predefined number of generations. After exchanging individuals in the communication phase, all of the subpopulations continue independently again. The above procedure operates iteratively until the termination criterion is met.

**Figure 3:**
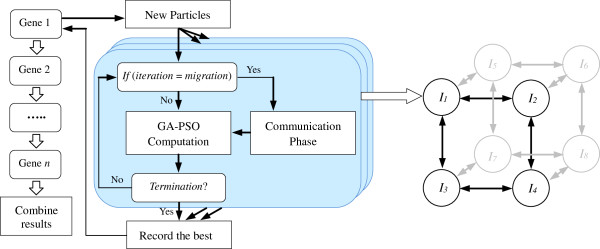
The parallel model of our iGA-PSO approach.

To implement the above model, a binary *n*-cube topology is configured in which each cube node corresponds to one of the networked computers. In this model, migration occurs only between immediate neighbors along different dimensions of the hypercube, and the communication phase involves sending a certain number of the best individuals of each subpopulation to substitute for the same number of the worst individuals of its immediate neighbors at regular intervals. Figure  3 illustrates the concept of the model and the operations with an example (*n* = 3).

### Parallelizing iGA-PSO on a cloud computing framework

**Figure 4:**
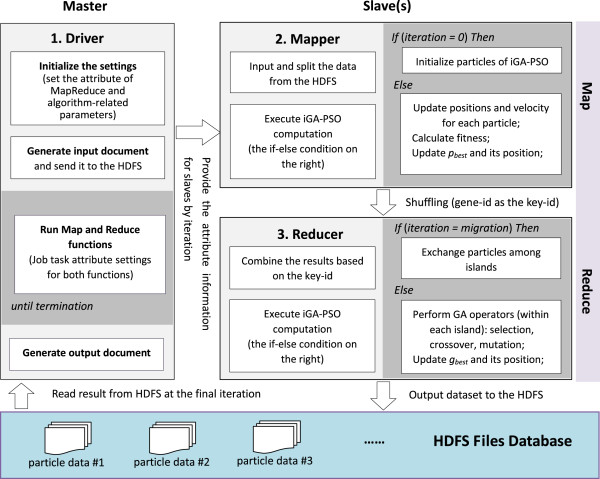
Overview of the proposed framework: iGA-PSO with the Hadoop MapReduce model.

To utilize the above parallel model to solve the problem of high computational load, we implement our iGA-PSO algorithm with the MapReduce programming model on the Hadoop cloud computing framework. As mentioned before, we have to decompose the original problem and parallelize the algorithm (i.e., iGA-PSO). Then, we also have to arrange different operations of the algorithm for the Map and Reduce modules, so that the framework can distribute the computation accordingly. Figure  4 depicts the work flow of how to transfer the parallel iGA-PSO to the MapReduce framework for inferring a decomposed S-system model. As shown, in this application the operations of iGA-PSO cooperate with each other in the map and reduce phases. This parallel implementation mainly includes four user-defined objects (specified by the Hadoop MapReduce working model) that are described below. More technical details about the user-defined objects are described in a Additional file 1.

The first user-defined object is the map method (designed for the Mapper). This object allows the algorithm to execute the operations independently without waiting for others, and thus carries out the parallel computing. In the decomposed S-system model, the set of parameters for each individual gene can be independently inferred, and this characteristic matches the purpose of using Mappers for problem solving. In our iGA-PSO algorithm, some operations for optimizing the parameters that correspond to a specific gene can be executed concurrently (including the computation of updating a particle’s position and velocity, the fitness evaluation for each particle, and the determination of the best-so-far result for each particle (i.e., *p*_*best*_)). They are thus arranged to be executed in the mapper phase. Such an arrangement means creating some input files by following the format specified by the MapReduce framework (see the third object below). According to the files, this framework will distribute particles (when they are to perform the operations mentioned above) to different Mappers to achieve the parallelism automatically.

The second user-defined object is the reduce method (designed for the Reducer). In contrast to the above map method, this method deals with the operations that involve results generated by others. Some operations in our iGA-PSO algorithm need to wait for others and they are thus allocated in the reduce phase (including the execution of selection, crossover, and mutation in the GA procedure, the operations of migration among different islands in the parallel model, and the determination of the global best result, i.e., *g*_*best*_). In the MapReduce framework, the particles have to be re-distributed to the computational nodes (working as the Reducers in this phase) before they start to perform the above operations. Here, we group the particles that deal with the same genes together, and each group of particles is dispatched to a Reducer.

**Figure 5:**
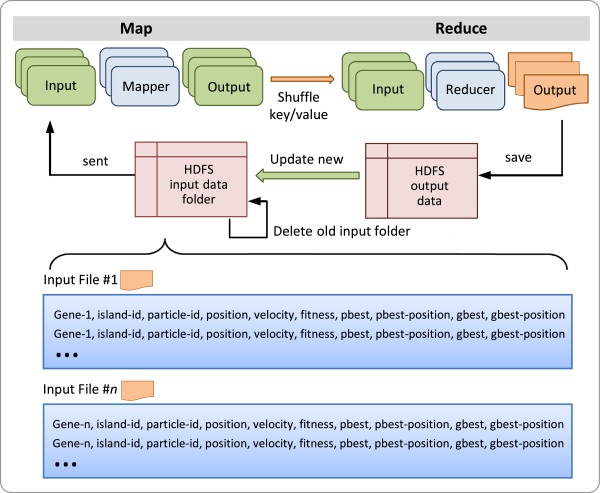
The proposed input/output file format for the Mapper and Reducer.

The third and the forth user-defined objects are input and output formats respectively. The former will be read and processed by the map method; the latter, transferred to output records by the reduce method. This is for creating a data string for each particle to specify its role and record the relevant information (see the input file format in Figure  5), so that different operations regarding to the iGA-PSO algorithm can be performed correctly on the MapReduce framework in different phases. In this study, there are two types of data defined in each string: the identifier for the recognition purpose in the MapReduce process; and the particle state for indicating the states of the particles used in the iGA-PSO algorithm). The first type of data includes three different identifiers: (1) gene-id, which is assigned to a particle at the initialization phase to indicate which gene this particle is responsible for, and it is used to distribute the particle to a specific Reducer in the reduce phase; (2) island-id, that each particle is assigned with; and (3) particle-id, which is an identification number of a particle and is used for tracing the computational result of the particle. The second type of data indicates the most recent particle states: the position, velocity, and fitness, the *p*_*best*_-position of a particle, and the *g*_*best*_-position of the swarm. Here, the fitness means the performance of this particle, and the other entries describe the particle states (as described in the above iGA-PSO). With the above two types of data, this MapReduce framework can activate the corresponding operations in the map and reduce phases, respectively. Details about the control flow and the data format settings of the MapReduce model for this application are described in the Additional file 1.

As shown above, the proposed approach can efficiently distribute the iGA-PSO algorithm to the map and reduce phases. In this way, our approach can fully exploit the computational power of the cloud computing system to perform parallel computation for network inference. The proposed framework is able to remedy the scalability problem and be used to infer large gene networks.

## Results

To assess the performance of our parallel iGA-PSO in a cloud computing environment, we used the well-known open-source software *GeneNetWeaver* 3.1 (GNW 3.1, [37]) to create several datasets. GNW was designed for generating *in silico* benchmarks of gene expression profiles by extracting network modules from prior *in vivo* studies (such as *S. cerevisiae*[38,39]) and connecting/expanding these modules to form test networks. In other words, each benchmark is composed of hundreds of genes (that form the real biological sub-networks), and the network can be inferred by computing algorithms without performing an *in vivo* experiment in advance. According to the GNW tutorial, the benchmarks used in the competition of gene network inference in DREAM challenges were created in this way [40].

In the experiments, four datasets were generated from the software GNW 3.1. They included a 25-node (dataset 1), a 50-node (dataset 2), a 100-node (dataset 3), and a 125-node (dataset 4) yeast *S. cerevisiae* sub-networks. Then we used this software to produce the time series data for each test network. In the generation of time series data, we left most of the settings at the default values suggested in GNW 3.1 as in DREAM4, for example, the perturbation of the time series, the model of noise in microarray, and the normalization after adding noise. To increase the complexity of the network modeling for rigorous verification, the duration of the time series and time points were approximately 1.5 times of the default settings (changing from 21 to 31 time points). The main reason for generating datasets by GNW 3.1 rather than directly taking the datasets of DREAM challenges lies in that our evaluation procedure requires a set of different sizes networks with certain scale differences to compare the corresponding speed-up rates. As far as is known, there has not been any work that infers S-systems to characterize the gene networks used in the DREAM challenges, so no results of the same type are yet available for performance comparison.

With the above datasets, two phases of experiments were arranged and conducted. The first phase was to evaluate the performance of our network inference method without (i.e., GA-PSO) and with the island model (i.e., iGA-PSO for virtual parallelism), by running the code on one sequential machine. In the second phase of the experiments, the iGA-PSO method was implemented on the Hadoop MapReduce framework to speed up the gene network reconstruction (i.e., real parallelism), and the amount of time required to complete a run of network modeling using different methods was compared.

### Performance of the proposed algorithm on a sequential machine

The first experimental phase involves examining whether the coarse-grained parallel model can further improve the search quality of the original method. Therefore, for the above four datasets, the optimization algorithm with two different settings was used to infer the networks. The first setting was the original GA-PSO (without the island model), which can also be regarded as the iGA-PSO with only one island of the population. The second setting was the GA-PSO with the island model in which different numbers (2 and 4 in the experiments) of subpopulations were used. In the experiments, the migration procedure was activated every *m* iterations (10 or 20 in the experiments), and the number of migrants to be exchanged was 5% of the sub-population. For each subpopulation, the migrants were chosen randomly from the top 20% particles of a sub-population to replace the worst 5% of its neighbors. These values were chosen based on a small pilot study.

Twenty independent runs of 200 iterations were conducted, in which the population size was 8,000 for the first dataset, and 10,000 for the other datasets. As mentioned above, the proposed approach includes PSO and GA parts, and a certain amount of individuals are randomly generated to form the parent pool for the creation of new individuals (see Figure  2). In the experimental runs, we chose a proportion of 70% of the individuals for PSO improvement and a static rate of 10% for random individuals (i.e., *p* was 0.7 and *r* was 0.1). Table  1 shows the results: the mean (i.e. Avg in the table), the best and worst performance of all runs; and the standard deviation for each set of experiments are listed. As seen, the method that involved working with a parallel model (i.e., iGA-PSO) outperformed its original form with regard to the results of the average, standard deviation, and best and worst fitness values for all four datasets. In addition, among the settings for iGA-PSO, the combination with island number 4 and migration interval 10 gave better results than the other combinations.

**Table 1:** Results obtained by the proposed algorithms with different settings for dataset 1 to 4

		**GA-PSO**	**iGA-PSO (*i*** **= 2)**		**iGA-PSO (*i*** **= 4)**	
	**Migration ( *m *)**	**-**	***m*** **= 10**	***m*** **= 20**	***m*** **= 10**	***m*** **= 20**
25 genes (dataset 1)	Avg	0.1437	0.1312	0.1246	0.1195	0.1240
	Best	0.1256	0.1117	0.1113	0.1026	0.1083
	Worst	0.1678	0.1516	0.1429	0.1311	0.1460
	SD	0.0149	0.0103	0.0085	0.0107	0.0093
50 genes (dataset 2)	Avg	0.2288	0.2031	0.2098	0.1944	0.2002
	Best	0.1813	0.1572	0.1606	0.1519	0.1515
	Worst	0.2516	0.2305	0.2267	0.2158	0.2254
	SD	0.0187	0.0174	0.0156	0.0147	0.0180
100 genes (dataset 3)	Avg	0.3947	0.3759	0.3765	0.3656	0.3711
	Best	0.3570	0.3304	0.3501	0.3337	0.3411
	Worst	0.4571	0.4067	0.4140	0.3957	0.3961
	SD	0.0272	0.0168	0.0176	0.0148	0.0160
125 genes (dataset 4)	Avg	0.2275	0.2197	0.2216	0.2177	0.2210
	Best	0.2111	0.2043	0.2053	0.2056	0.2087
	Worst	0.2497	0.2383	0.2468	0.2299	0.2374
	SD	0.0105	0.0096	0.0112	0.0068	0.0087

The results in Table  1 indicate that the parallel model can successfully enhance the performance of the original network inference method. This is consistent with other island-based EA works. It is because that using multiple subpopulations can effectively keep groups of particles evolving separately in different islands for some pre-defined generations, and this strategy has successfully maintained the diversity of each group of particles. Hence, each island would not be dominated by the elite particles in other islands. The algorithm can then prevent the so-called premature situation (meaning converging too fast) and can explore more regions of the solution space to find better solutions.

**Figure 6:**
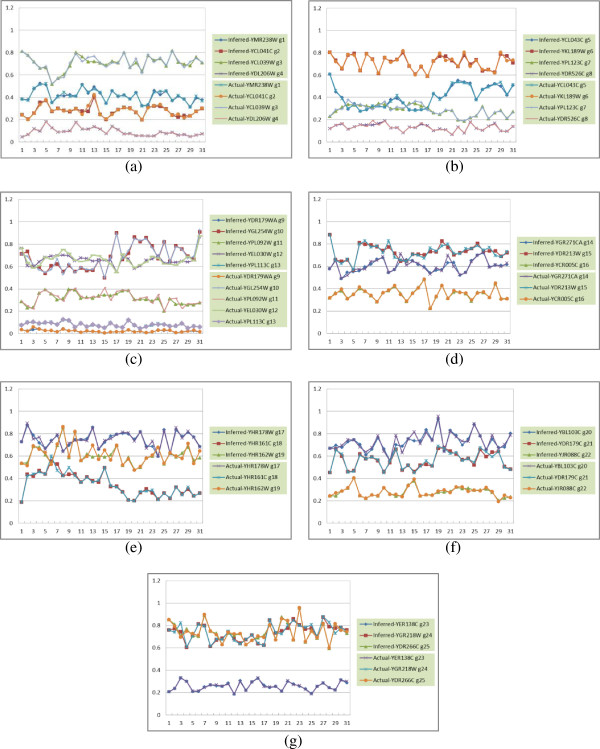
The inferred and the actual network behaviors of the 25-gene sub-network (dataset 1); they are split into seven sub-figures (a), (b), (c), (d), (e), (f), and (g).

**Figure 7:**
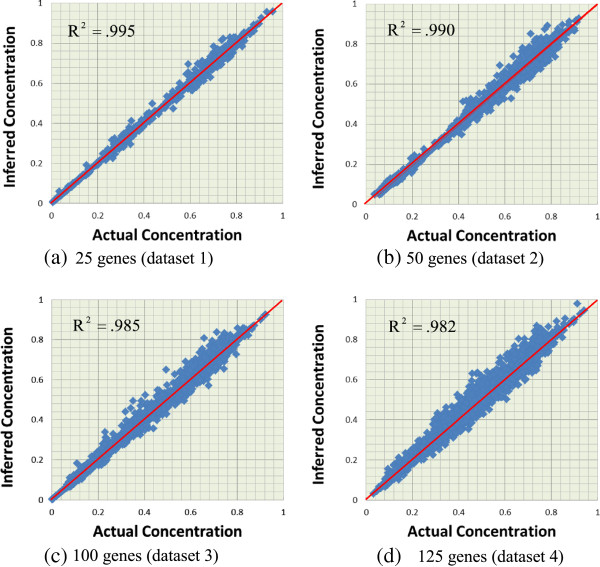
**The scatter plots for the relationships between the inferred and the actual concentration of the four datasets.** Sub-figures **(a) (b) (c) (d)** are for datasets 1, 2, 3, and 4, and the plots contain 775, 1,550, 3,100, and 3,875 dots, respectively.

The inferred (by iGA-PSO) and actual network behaviors are compared. Figure 6 shows the results of the 25-node network as a representative, in which the network was inferred in a decomposed way (i.e., gene by gene). Here, the *x*-axis represents the time points and the *y*-axis is the concentration levels of genes. To present the results clearly, in this figure we divided the results for this dataset into several sub-figures. As can be observed, very similar network behaviors were inferred for genes in this dataset. To provide a holistic evaluation for all datasets described above, we produced a scatter plot for each dataset to illustrate the inferred versus real expression values for all genes and all time points. Figure  7 presents the results for all the four datasets. In the figure, the *x*-axis and *y*-axis are the concentrations of the actual and inferred networks respectively, and the coordinate of each data dot represents the concentration of a gene at some time point. Thus, the diagonal of the scatter plot indicates the perfect data fitting. As shown in Figure  7, the data dots all locate at around the diagonal of each sub-figure, and these results confirm the performance of our iGA-PSO approach.

To investigate the effect of virtual parallelism (i.e., running the island model algorithm on a sequential machine) in more detail, we collected the results to analyze the computational cost of obtaining a successful solution using the methods with different subpopulations. Here, a "try until success" recursive strategy is used, in other words, independent experimental runs were performed sequentially until a successful run was obtained. In our network inference application, a successful run means that the solution obtained from the run has a fitness value that is lower than a threshold (measured by the evaluation function). Based on the above criterion, we summarize the results of different methods and present them in Table  2.

**Table 2:** The number of successful runs and the computational cost of the different methods

	**Dataset 1**			**Dataset 2**			**Dataset 3**			**Dataset 4**		
num. of population	1	2	4	1	2	4	1	2	4	1	2	4
population size	8000	4000	2000	10000	5000	2500	10000	5000	2500	10000	5000	2500
num. of successful runs	12	19	20	8	17	20	7	15	16	10	14	17
Expected Cost (*C*)	1.69	1.10	1.05	2.44	1.22	1.05	2.75	1.38	1.29	2	1.47	1.22

Given the chance of success from running a single experiment and the cost of running a single experiment, the recursive strategy described above allows us to calculate the expected computational cost *x* for a certain method using the following equation:

(10) $$x=C\times P+\left(C+x\right)\times \left(1-P\right)$$

In this equation, *C* is the computational cost of running a single experiment with this method, and *P* is the probability of obtaining a successful run with this method (the experiments are probabilistically independent). By solving this equation, we can obtain *x* = *C*/*P*. Suppose that we have conducted a few independent runs in which *M* of them succeeded and *N* of them failed; then, the distribution of the probability of success *P* can be described as ( [41]):

(11) $$f\left(P\right)=\frac{P^{M}{\left(1-P\right)}^{N}}{B\left(M+1,N+1\right)}$$

in which B(∙,∙) is the Beta function. Thus, we can calculate the expected cost of *x* as:

(12) $$\begin{array}{ll} \;E_{p}\left[x\right] & =\int_{0}^{1}\frac{C}{P}\times \frac{P^{M}{\left(1-P\right)}^{N}}{B\left(M+1,N+1\right)}\text{dP}\\ & =\frac{C}{B\left(M+1,N+1\right)}\int_{0}^{1}P^{M-1}{\left(1-P\right)}^{N}\text{dP}\\ & =C\times \frac{B\left(M,N+1\right)}{B\left(M+1,N+1\right)} \end{array}$$

By the definitions *B*(*m*, *n*) = *Γ*(*m*)*Γ*(*n*)/*Γ*(*m* + *n*) and *Γ*(*x* + 1) = *xΓ*(*x*), the above result can be simplified to:

(13) $$E_{p}\left[x\right]=C\times \frac{M+N+2}{M+1}$$

With this estimation approach, if we assume that the cost of conducting a run using the method of one population of 8000 individuals is *C*, and for this method we have *M* = 12 and *N* = 8, then the expected computational cost of obtaining a successful run with this method is *C* × 22/13 (= 1.69*C*). Similarly, for the method with two sub-populations of 4000 individuals, *M* = 19, *N* = 1, the cost of a single run is *C* (the cost for exchanging individuals is relatively small and ignored); thus, the expected cost of this method is *C* × 22/20 (= 1.1*C*). In the same way, we can estimate that the expected cost for the method with four subpopulations of 2000 individuals is 1.05*C*. The estimated costs for all of the datasets are listed in Table  2 (assuming that the run with one population is *C*). These results indicate that using iGA-PSO can indeed yield successful solutions with relatively less computational effort.

### Performance of the proposed method on parallel machines

After investigating the performance of the proposed algorithms (GA-PSO and iGA-PSO) running in a sequential manner for gene network modeling, in the second phase, we conducted two suites of experiments to examine how our iGA-PSO method can be performed in a parallel computing environment to speed up the computation in practice. The iGA-PSO was implemented on the Hadoop MapReduce framework and was executed on different computer clusters for verification. The first suite of experiments was conducted at one of the personal computer laboratories in our university computer center, and the second suite was conducted in a commercial cloud computing environment. In the campus computing laboratory, two sizes of computing clusters were arranged as slave machines, including 20 and 25 PCs (due to the hardware limitations), and one extra machine was set up as the master. These machines have the same hardware/software specification: 3.0 GHz Intel Core 2 Duo E8400 CPU, 4GB DDR2 667 RAM, and Linux CentOS 6.2 (64-bits) or Ubuntu 11.10 (64-bits) operation system with Hadoop 0.20.0 version. The transmission speed of the local area network is 1,000 Mbps. We also ran the sequential version of GA-PSO and iGA-PSO on a single node for a baseline comparison.

It is notable that the main purpose of this study is to provide a practical framework to speed up the computation so that large biological regulatory networks can be inferred, rather than to compare different algorithms or to perform optimal parameter settings. Therefore, in this experimental phase, we considered the island number to be 4 and migration interval to be 10 (the parameter combination with the best performance as shown in the previous section) in the experimental runs of real parallelism. Here, we focused on the data sets that had more gene nodes (i.e., 50, 100, 125 nodes). Because of the large amount of computational time needed, in these runs, the population size was reduced to 5,000 and the iteration number was 200 iterations.

Table  3 show the results; the arrangement of computational nodes (i.e., master/slave), the time (in minutes) spent for the experimental runs, the speed-up rates, and the average fitness value per gene obtained from the runs are all listed. These results indicate that the implemented Hadoop MapReduce framework can indeed reduce the experimental time that is required to infer gene networks, which makes it possible to infer large networks with more nodes. From the table, we note that the original GA-PSO (which takes 923 minutes to complete a run for dataset 2, 3,687 minutes for dataset 3, and 5,788 minutes for dataset 4) is slower than the sequential iGA-PSO (which spends 908, 3,137, and 4,927 minutes for datasets 2, 3, and 4, respectively), which, in fact, required extra computational effort to address the inter-island communications in the experiments. After further inspection, we found that the reason is that the iGA-PSO method used 4 islands with populations of 1,250 to handle the computation, which reduced the sorting time (e.g., to sort the possible solutions and find the best members) that is needed in the GA part of the hybrid algorithm. Therefore, to make a more objective comparison of the speed-up rate between the sequential and parallel versions of the proposed algorithm, we chose to use the run time of the sequential iGA-PSO as the baseline record.

**Table 3:** Results for datasets 2, 3, and 4 by running the experiments in the computer center

	**Algorithm**	**Sequential**	**Sequential**	**Parallel**	
		**GA-PSO**	**iGA-PSO**	**iGA-PSO**	
50 genes (dataset 2)	Master / Slaves	1 / 0	1 / 0	1 / 20	1 / 25
	Time cost (mins)	923	908	218	176
	Speed-up	-	1	4.1651	5.1591
	Fitness value per gene	0.2876	0.2494	0.2593	0.2562
100 genes (dataset 3)	Master / Slaves	1 / 0	1 / 0	1 / 20	1 / 25
	Time cost (mins)	3,687	3,137	496	411
	Speed-up	-	1	6.3246	7.6326
	Fitness value per gene	0.4567	0.4225	0.4165	0.4114
125 genes (dataset 4)	Master / Slaves	1 / 0	1 / 0	1 / 20	1 / 25
	Time cost (mins)	5,788	4,927	702	595
	Speed-up	-	1	7.0185	8.2807
	Fitness value per gene	0.2822	0.2651	0.2670	0.2667

**Figure 8:**
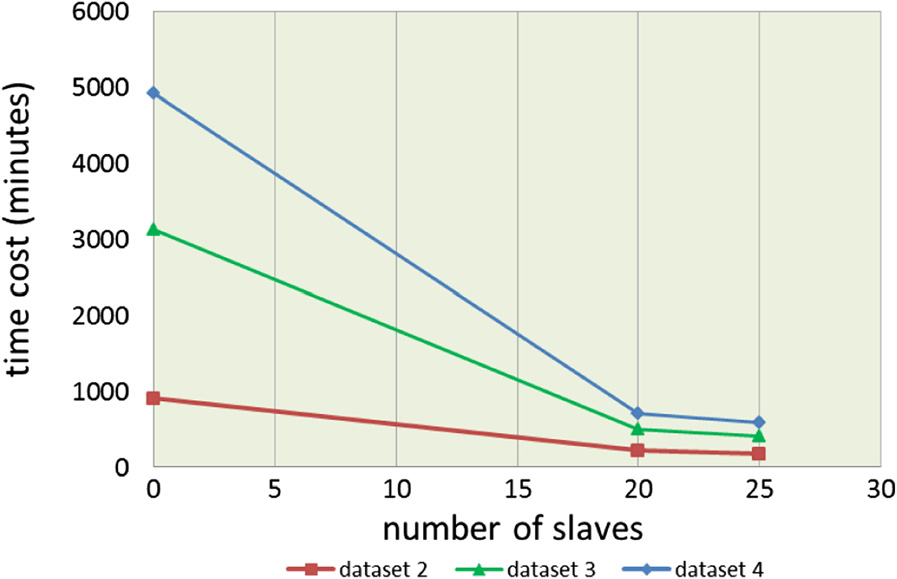
The time cost curves of different sets of experiments conducted in the university computer center.

Figure  8 illustrates the time cost curves of the results listed in Table  3. As shown in this figure, the parallel iGA-PSO on 20 slaves is approximately 4.17 times faster (i.e., from 908 down to 218 minutes) to 7.02 (i.e., from 4,927 down to 702 minutes) than the sequential iGA-PSO (without any slave) for the three datasets, and the 5.16 ~ 8.28 speed-up rates can be obtained for the experiments with 25 slaves. Overall, the best speed-up performance that the framework can achieve here is for the cases of running the parallel algorithm on 25 slaves to infer networks for dataset 4. In other words, the parallel iGA-PSO is 8.28 times faster than the sequential iGA-PSO on a single machine.

After running the proposed iGA-PSO method on the Hadoop MapReduce framework to prove its correctness and confirm its performance, we conducted the second suite of experiments to further evaluate this parallel method in the cloud. Among the suppliers of cloud computing services, the Amazon Elastic Compute Cloud (i.e., Amazon EC2) is one of the most famous and largest cloud computing and storage providers. It provides a basic but versatile computing environment at a low rental cost, including scalable CPUs, memory (RAM), disk storage, and several operating systems. In addition, the cloud services also allow users to expand or eliminate computing nodes and to pay only for the activated nodes. Fusaro *et al*. drew our attention to how projects in biomedical informatics were deployed on the Amazon cloud service and listed the pricing structure from Amazon EC2 for the CPUs and storage [42].

Similarly, one of the telecommunication service providers, Chunghwa Telecom, which is the largest cloud computing supplier in Taiwan, offers application developers ideal computing resources (called *hicloud*, http://hicloud.hinet.net/). Comparing their pricing strategies, *hicloud* is cheaper than Amazon EC with regard to the matching on-demand packages, which fulfilled our requirements. Because using a reverse engineering approach to construct GRNs is a CPU- and memory (RAM)-consuming task, especially in the modeling of large networks (e.g., with more than 100 genes), we need efficient CPUs, a large memory capacity, and high-speed intranet data transfer to achieve this task. Therefore, considering both the price (each node costs $0.128 per hour)^a^ and the performance of the on-demand package (each *hicloud* node is equipped with a 2.0 GHz 2007 Xeon processor (4 virtual cores), 8 GB of RAM, 100 GB of disk storage, and Linux Ubuntu 11.10 (64-bit)), we chose *hicloud* as the cloud environment for this suite of experiments.

**Figure 9:**
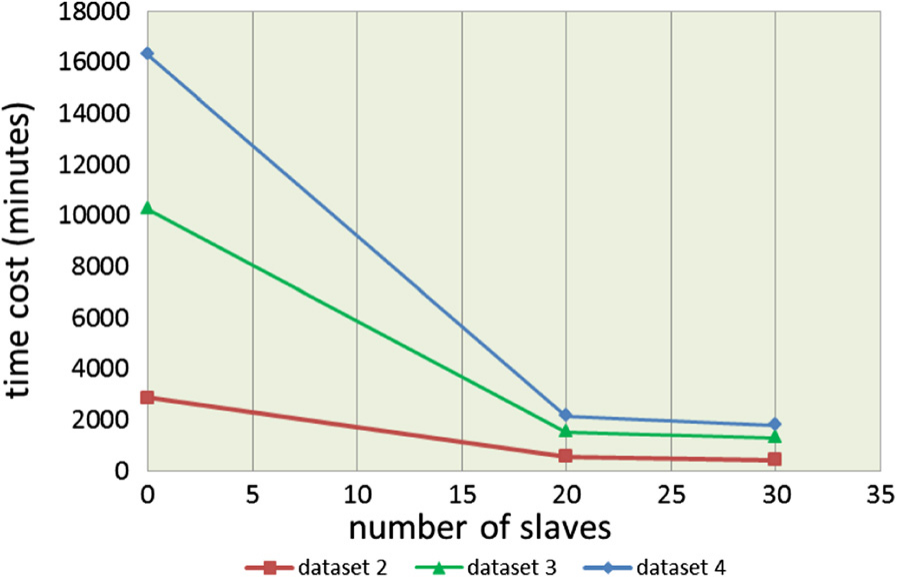
**The time cost curves of different sets of experiments on *hicloud*.**

With the *hicloud* computing clusters, we conducted the experimental runs for datasets 2, 3, and 4, to evaluate the proposed approach. For these datasets, 20 and 30 slave nodes were used to perform the parallel computation. Similar to the first suite of experiments, the sequential versions of GA-PSO and iGA-PSO were run for a performance comparison. In the experiments, the population size was 10,000, and each run lasted for 200 iterations. The results are presented in Table  4. As is shown, the speed of running the parallel iGA-PSO on 20 slaves was approximately 4.9 ~ 7.6 times faster than that of the sequential iGA-PSO in assessing the three datasets; and when the number of slave nodes increased up to 30, even better performance could be obtained. To sum up, compared with the sequential iGA-PSO, the best case is to run the parallel iGA-PSO with 30 slave nodes for dataset 4, that yields a 9.1 times speed-up performance. Figure  9 illustrates the time cost curves of the results listed in Table  4. It shows that the sequential GA-PSO and iGA-PSO took much longer than their parallel versions running in the cloud computing environment. All confirms the efficiency of our framework.

**Table 4:** **Results for datasets 2, 3, and 4 by running experiments on *hicloud***

	**Algorithm**	**Sequential**	**Sequential**	**Parallel**	
		**GA-PSO**	**iGA-PSO**	**iGA-PSO**	
50 genes (dataset 2)	Master / Slaves	1 / 0	1 / 0	1 / 20	1 / 30
	Time cost (mins)	3019	2898	591	465
	Speed-up	-	1	4.9036	6.2323
	Fitness value per gene	0.2345	0.2115	0.2140	0.2076
100 genes (dataset 3)	Master / Slaves	1 / 0	1 / 0	1 / 20	1 / 30
	Time cost (mins)	11342	10284	1526	1316
	Speed-up	-	1	6.7392	7.8146
	Fitness value per gene	0.3843	0.3520	0.3642	0.3582
125 genes (dataset 4)	Master / Slaves	1 / 0	1 / 0	1 / 20	1 / 30
	Time cost (mins)	18019	16323	2152	1792
	Speed-up	-	1	7.5850	9.1088
	Fitness value per gene	0.2358	0.2056	0.2195	0.2195

## Discussions

**Figure 10:**
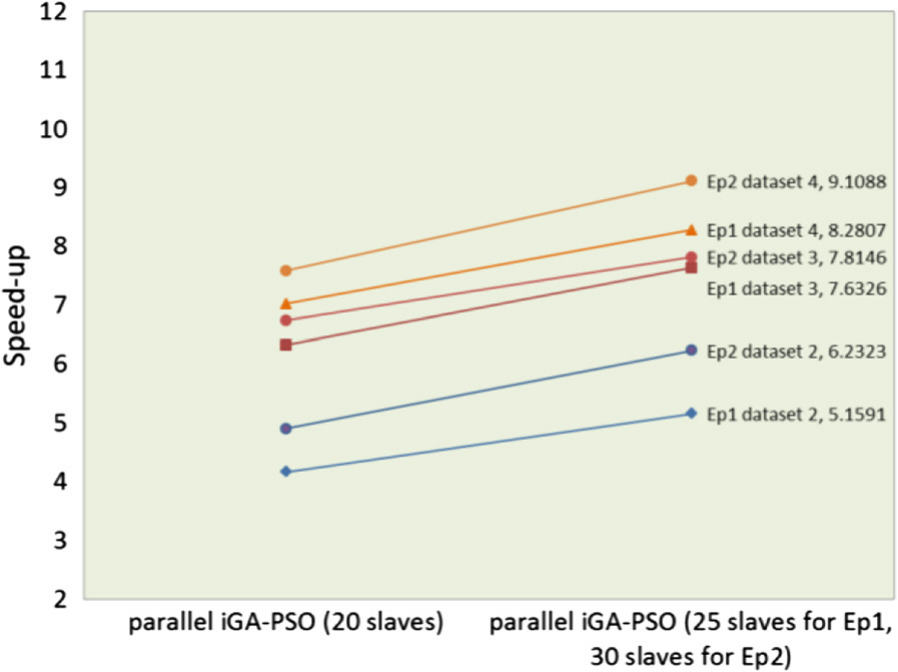
The comparison of speed-up curves of the first suite (Ep1) and the second suite (Ep2) of experiments.

The results obtained from the above two suites of experiments show that the larger the dataset that the proposed algorithm is applied to, the better the speed-up rate it can provide. Moreover, it can be observed from the results that the second suite, with a population size of 10,000, was slightly faster than the first suite, with a population of 5,000, in the computing environment of 20 slaves for all of the datasets (see Figure  10). By carefully recording and analyzing the computation process, we found that this result arises mainly because Hadoop MapReduce always requires some computational effort to initialize the framework, activate a job, transmit a job task via the intranet, and write/read the results into/from the HDFS. If a Mapper does not spend relatively more computation time (compared with the time spent on the system maintenance, as indicated above) on the major part of the application task at each iteration (i.e., less than 30 seconds in our case), the speed-up effect will be limited.

In the current era, scalable parallel computing allows application developers to exploit all of the degrees of freedom of in linear scaling to solve a time-consuming problem [43]. In this new epoch, it always comes to a critical issue that if a sequential computational problem can be divided into several parallel sub-problems (as shown in above section), then how to judge the speed-up performance obtained? Ideally, the computation in Hadoop MapReduce operates in parallel and different parts of the framework have no mutual influences with others. This way, the speed-up rate can increase linearly in proportion to the number of computational nodes. Taking our application as an example, a 20-node cluster should give a nearly 20 times speed-up rate in a completely independent MapReduce environment. Nonetheless, a particular portion of serial overhead and the parallel environment-enabling cost must be taken into account and which slows down the experiments in practice.

As the Amdahl’s Law described [44], because the non-parallelized portion of a task restricts the speed-up performance, a linear speed-up rate is in fact difficult to achieve. According to Amdahl, the inherent non-parallelized part of a computational task creates an inevitable constraint on the speed-up rate against the parallel processes available. The magnificent argument for the efficiency and limit of parallel computing has been widely adopted and discussed. To date, the most well-known version of Amdahl’s law can be expressed as follows:

(14) $${\text{Speed‒up}}_{\text{rate}}\left(f,S\right)=\frac{1}{\left(1-f\right)+\frac{f}{S}}$$

Where *f* is the proportion of a problem that can be divided into parallel computing, and (1- *f*) means the proportion that cannot be parallelized (e.g., the non-parallelized task, or the other execution processes). Ideally, the maximum acceleration lies in *S*, meaning that *f* can be accelerated *S* times by using *S* parallel processors (i.e., the slaves on Hadoop). For example, suppose modeling a gene network took 200 minutes by running a sequential iGA-PSO, and there was 5% (i.e., 10 minutes) of computation belonging to the non-parallelized task. Under such circumstance, no matter how many slaves we applied and devoted to the parallel computing, the remaining 95% (190 minutes) led the maximum acceleration up to 20 times at most (the minimum time–cost cannot be less than 10 minutes). Similarly, if a task needed to take 10% of computation for running the mechanism of cloud computing and for executing the non-parallel processes, then the maximum speed-up rate was limited to 10 times.

In this study, the best acceleration rate we can obtain in the experiments is 9.1 (from the case of inferring gene network for dataset 4 with 30 computational nodes in the second suite of experiments, see Table  4). It means that we have parallelized 89% (1-(1792/16323)) of the whole modeling task, and only remained 11% of the original execution time for the non-parallelized overhead and for the cost of maintaining the Hadoop MapReduce framework.

In addition to the speed-up rates, other issues related to the performance of the network model are worth mentioning. Considering the use of a decoupled S-system model, some studies have pointed out the model’s characteristic, and the pros and cons of decomposing the original ODEs model (e.g. [4,45]). In short, the decoupling strategy proposed by [4] enables us to infer one differential equation at a time to match the target expression data. Then, after the parametric solution for each single differential equation is provided, two strategies for deriving a final solution set are suggested.

The first strategy is to combine all single decoupled solutions into one and then to depict the entire network system directly. This strategy is suggested when every expression profile was precisely estimated. It means that the errors of the optimal solutions generated by the decoupled approach will not have a significant effect on the network behaviors when all the solution sets are assembled to form a coupled model [17]. However, when the solution sets obtained from the decoupled models cannot reproduce the expression profiles correctly, the second strategy, a recoupling procedure, has to be carried out. It arranges all the decoupled parametric sets to form a coupled S-system model, and then uses this model as the initial parameter population to continue the optimization process. This strategy, nevertheless, is computationally expensive as reported in [45]; an alternative approach has been suggested that is to turn to a cooperative evolutionary method to facilitate the recoupling process [17]. After evaluating the estimated expression profiles shown in Figure 7, in which all the estimated datasets have very high R-squared values (all above 0.98), we adopt the first strategy in this work.

To evaluate the performance of adopting the S-system model to infer both the gene expression profiles and the corresponding network structure, we propose another fitness function in the Additional file 1. This fitness function concerns not only the network behaviors but also the sensitivity and specificity rates. The real positive and negative outcomes obtained from the real world resources are used to help the new fitness function to evolve more reasonable structural parameters while optimizing the expression profiles at the same time.

We have also selected one dataset of the DREAM challenges and conducted a set of experiments to compare our approach with other well-known reverse engineering methods. To be specific, in this set of experiments, the DREAM4 multifactorial network 1 with 100 nodes was used, and two well-known inference algorithms (GENIE3 [46] and TIGRESS [47]) were chosen for comparison. Our approach was performed with two fitness functions: (a) the MSE function, and (b), the fitness function described in Part C of the Additional file 1. The ensuing precision and the recall rates for fitness functions (a) and (b) are (91.57%, 5.08%) and (97.93%, 10.6%), respectively. These results reveal that if some useful structural information is integrated into the fitness function, the S-system model can be derived to optimize both the network’s behaviors (the profiles) and the structure. Note that the trade-off between optimizing the system dynamics and network structures always exists [48].

Before comparing our structure correctness rates to the results obtained by the state-of-the-art structural prediction algorithms, two concerns need to be mentioned. First of all, when using the MSE as the fitness function in inferring the S-system, we can only claim that the network profiles have been reconstructed properly if the fitness value is lower than an acceptable threshold. There is no guarantee that the structural correctness can be as acceptable as the numerical results. Therefore, it is helpful to apply some extra strategies to ensure the correctness of an inferred model. One promising way is to modify the MSE function: it means to include the structural information matrix to guide the search of genetic parameters. Through the constraints of the structural information, an outcome with correct network profiles and structure can be obtained.

The second concern is that the goal of our inference approach is different from that of the studies conducted for the DREAM challenges. They have focused on the prediction of the network structure. However, here, we rely on the structural information collected from the real world resources, so that we can use a classification matrix to guide the development of solution space. As a result, the correctness rate on the network structure thus depends on the available structural information. Most importantly, the inference method is presented to show how a network can be reconstructed to provide the required system dynamics and relative connection relationships among genes (but not for predicting a network structure of a general graph-based model). The computational model can be replaced by others.

After clarifying the primary differences between our approach and the predictive reverse-engineering methods, we are aware of the limitations of directly comparing our results to TIGRESS and GENIE3 methods. Therefore, by carefully examining and summarizing the results of the above two algorithms, we provide their precision and recall rates only for reference. Overall, if the precision is around 0.9, then its relative recall rate lies in the range of 0.1 to 0.15 for Dream4 networks [46,47].

## Conclusions

In this study, we have emphasized the importance of reverse engineering GRNs from gene expression profiles. Depending on the biological level to be studied, many models have been proposed to simulate GRNs, among which concrete models are more suitable for simulating biochemical processes realistically. To infer gene networks, we adopted a well-researched concrete model, the decomposed S-system model, to represent a network and to infer the relevant network parameters. Although this model has been simplified from its original form, the computational cost to infer such a model still grows linearly with the number of gene nodes that are involved. To overcome the scalability problem, we presented a practical framework that can efficiently determine network parameters and is scalable for inferring large-scale networks. In this framework, a hybrid GA-PSO optimization method was developed and parallelized for performance enhancement. To conduct the real parallelism to reduce the computation time, our parallel method was extended to work with the Hadoop MapReduce programming model and was executed in different cloud computing environments. Extensive sets of experiments and analyses have been conducted to evaluate the proposed framework. The results show that our approach can successfully infer networks with desired behaviors for real-world biological datasets. Most importantly, our approach can be used to infer large gene networks on the clouds, in which the Hadoop MapReduce framework has been shown to substantially reduce the computation time for the application here. Currently, we are investigating different ways to take both the fault tolerance ability and the computing performance into consideration and developing an even more efficient framework to infer networks with more nodes.

### Endnote

^a^The specifications of each node provided by Amazon EC2 are: (1) High-CPU Medium Instance with 2.5-3.0 GHz 2007 Xeon processor (2 virtual cores), 1.7 GB of memory, 350 GB of local instance storage. (2) High-CPU Extra Large Instance with 2.5-3.0 GHz 2007 Xeon processor (8 virtual cores), 7 GB of memory, 1690 GB of local instance storage. The High-CPU Medium package cost $0.185 per hour/node. The High-CPU Extra Large costs $0.740 per hour/node.

## Competing interests

The authors declare that they have no competing interests.

## Authors’ contributions

WL conceived the project, designed the algorithm, wrote a part of the manuscript, and made modification as well as final revision. YH undertook parts of the experimental implementation and wrote a part of the manuscript. WH set up the experimental environments and undertook parts of the experimental runs. All authors read and approved the final manuscript.

## Supplementary Material

Additional file 1 Part A: Decomposing a decouple S-system model onto the MapReduce Framework. Part B: Control flow and data format. Part C: Using structural knowledge in network inference. Click here for file
