# Supplementary material for: Designing a parallel evolutionary algorithm for inferring gene networks on the cloud computing environment
Source: BMC Syst Biol. 2014 Jan 16;8:5. doi: 10.1186/1752-0509-8-5 (PMC3900469; doi:10.1186/1752-0509-8-5)
Supplement: Additional file 1 — Part A: Decomposing a decouple S-system model onto the MapReduce Framework. Part B: Control flow and data format. Part C: Using structural knowledge in network inference. [file 1752-0509-8-5-S1.doc]

**Part A: Decomposing a decouple S-system model onto the MapReduce Framework**

The following walkthrough example explains in detail how to use the MapReduce method to perform computation for a decouple S-system model.

**. . .**

**. . .**

**. . .**

output file 1

Spilt file by line

Mapper

Gene 2

input file 2

Reducer

output file 2

Spilt file by line

Mapper

Gene 3

input file 3

Reducer

output file 3

Spilt file by line

Mapper

Gene *n*

input file *n*

Reducer

output file *n*

Spilt file by line

next generation

Reducer

Mapper

input file 1

Shuffling (gene-id as the key-id)

next generation

next generation

**Map** **Reduce**

Gene 1

Driver

The Master machine

next generation

2

1

3

4

5

Fig. S1: The computational flow of distributing the computation to the mappers and reducers in a single iteration.

Fig. S1 depicts the conceptual view of how the computational operations for the decomposed S-system model are distributed, and how they are executed in the MapReduce framework. Suppose the target GRN consists of 100 genes and there are 20 computing nodes available for running mappers and reducers (the nodes are reusable for two phases). When the user-defined program in the driver is activated, the driver starts to deploy the MapReduce process. Five main steps of the deployment occur in the following order:

First, the driver creates 100 input data files (i.e., *n* = 100 in this figure) and each file records the details (i.e. the computational information for the mapper and reducer) corresponding to one specific gene of the decoupled S-system model. According to the system settings of MapReduce, each file is performed by a computing node. This configuration is convenient for running the operations of a parallel evolutionary algorithm (EA).

Second, the driver then dispatches a number of job tasks to the mapper nodes. In this example, there are 100 genes to be inferred. Therefore, each node is responsible for 5 map tasks. Note that if the size of a single file exceeds the maximum memory capacity, e.g. 64 MB, the file will be divided into smaller tasks. In our application case, one file corresponds to a map task.

Third, a mapper node reads the file and divides the file line by line. Each line has a string format that contains all the information to be utilized for the parallel EA. The line (string) is regarded as the basic unit for a specific gene of the S-system model. After performing some computation specified by the user-defined program (e.g. the EA operations), a mapper saves the results in the local memory and sets gene-id as the *key-id* to be used by the reducer in the next step. The concept of using the key-id in MapReduce is that all information corresponding to a target gene is identified by the given key-id, so the key-id can be used by the reducers to retrieve the information of a specific gene. The results corresponding with the same key-id are then collected for the same reducer.

Fourth, once the computing nodes are idle, they become the reducer nodes. After a reducer groups the operations related to a specific gene, it starts to execute the user-defined program and output the results corresponding to the key value. Then, the reducer will continue to group the operations for the next key-id until output for all key-ids (i.e., gene-ids) have been produced in the reduce phase.

Fifth, the driver continues to designate job tasks for mapper nodes, if the above process needs to be performed iteratively. If not, the driver will summarize the final result of the complete model. In this example, the inferred results for the 100 genes are aggregated by the reducer.

In the process mentioned above, MapReduce takes care of almost all of the low-level details, including the data distribution, communication, fault tolerance, etc. one can therefore concentrate on the algorithms and define the map/reduce methods. Specifically speaking, the user only has to design four objects, which are categorized into two types of computational functions: map and reduce phases. The first two objects are for the map phase, including a map method and the input format with respect to the key-id/value pair per record that would be read and processed by the map method. Here, the key-id represents one individual gene in the S-system model and the value along with a key-id records the information for the user-defined computational operations, such as the parameters used in the parallel EA. The remaining two objects for the reduce phase include a reduce method and the output format that will be transformed into output records.

**Part B: Control flow and data format**

The operational flow of the MapReduce model can be described in detail as follows (see Fig. 4 of the manuscript). The driver module in the master machine is responsible for the iteration control of the iGA-PSO computation performed by the slaves. In other words, every iGA-PSO iteration starts a dispatching process once to distribute the relevant operations into the map and reduce phases for computation. The driver also sends the input document to the HDFS files database at the beginning and produces the overall output file from the HDFS at the end of the run. Specifically, in the Hadoop environment, the MapReduce process is initialized by the driver, which generates a “Job” and sends it to the Job Tracker. The Scheduler within the Job Tracker then produces two types of subtasks: MapTask and ReduceTask (to be executed in the *mapper* and *reducer*, respectively) and dispatches them to the slave machines. The iterative flow is achieved by the driver to create a new Job and repeat the above procedure.

Before sending a Job to the Job Tracker, the driver must create a Configuration object to store the parameter settings in HDFS (such as the particle number, the weights for updating the particle velocity, the migration rate, and so on). These parameters are saved in the HDFS folders and are shared between the mapper and reducer to ensure data correctness during the algorithmic computation. Because some parameter values (such as the weight of each particle) will be changed in each iteration and the computation results conducted in the mapper or reducer are subject to those values, the driver must designate the Configuration for each new Job. In this way, a newly created Job can conveniently read the parameters from the files, and the iGA-PSO code in the mapper and reducer phases can directly use the parameters to perform the corresponding computation afterwards.

Between the map and reduce phases, the data are shuffled (parallel sorted/exchanged between computational nodes). The shuffling mechanism groups the particles together according to which genes they are addressing (by using the gene-id as the key). In this way, all of the particles that correspond to a given key end up at the same reducer to perform the remaining operations that must rely on the results produced by others. Here, using gene-id as the key in shuffling can keep the particle distribution uniform and thus alleviate the problem of becoming overloaded. This problem often occurs in the implementation of evolution-based algorithms with the MapReduce model. It is caused by a situation in which the algorithm proceeds (converges), the same (close to optimal) individual starts to dominate the population, and all copies of this individual are sent to a single reducer. In other words, the distribution of the computation becomes unbalanced, and the efficiency of the parallelism decreases as the algorithm converges. As a result, the algorithm will require more iterations to derive the final solution.

As can be observed from the data format used in the proposed approach (see Fig. 5 in the paper), the particles play central roles in conducting the computation. Specifically, at each MapReduce stage (iteration), we create a data string for each particle so that the Hadoop environment can handle the overall computational workflow smoothly. In this study, as mentioned in the paper, the data recorded in the string is categorized into two types. The first type of data is defined as the identifiers for the shuffling procedure; i.e., gene-id, island-id, and particle-id, which indicate the gene that a particle is addressing, the group that the particle belongs to, and the performance rank of the particle, respectively. The second type of data is defined to indicate the particle states; i.e., the position, velocity, and fitness, the *p_best_*-position of this particle, and the *g_best_*-position of the swarm.

With the above settings for control flows and data format, the driver module can control the two parts of the iGA-PSO without continuing to read the HDFS iteratively (as shown in Fig. 4 of the manuscript). This goal is achieved through the procedure of periodically updating the Job’s input/output paths (files) recorded in the HDFS. After the MapReduce process has been performed once, the driver changes the path of the input file to be that of the output file, to use the computational results that were obtained from the previous iteration. The driver simply passes the corresponding path to a mapper to perform the relevant computation; and, thus, saves much of the I/O effort by only writing/reading the HDFS at the first/final iteration.

**Part C: Using structural knowledge in network inference**

To demonstrate how the structural knowledge can improve the correctness of the network topology, we use two different fitness functions to conduct a set of experiments for comparison: the original MSE function, and the other fitness function. The new fitness function includes two major parts as follows:

$$f_{obj}(i)=\alpha\cdot MSE(i)+(1-\alpha)\cdot StrEval(i),\text{ for }i=\text{ 1, 2, 3, ..., }N$$

The first part, *MSE*(*i*), is used to derive the correct network behavior, whereas the second part, *StrEval*(*i*), is used to maximize the structural accuracy that measures the difference between the structure suggested by the pre-defined real positive and negative outcomes and that of the inferred model. The weighting factor *α* is used to decide the importance of the two issues to be considered (i.e., the gene expression profiles and the network structure). In the above function, there are two sub-terms included in *StrEval*(*i*), *sensitivity* and *specificity*, as described below.

$$StrEval(i)=sensitivity(i)+specifictity(i)$$

The two sub-terms are ratios of the binary classification result. In this equation, *sensitivity*(*i*)$\in$[0, 1] is the *true positive* rate. It indicates how many kinetic orders belonging to gene *i* follow the suggestion that a plausible connection (a *true positive* connection) should exist between gene *i* and other genes *j*. In contrast, *specificity*$\in$[0, 1] is the value of the *true negative* rate, which means how many kinetic orders belonged to gene *i* are negative connections.

The above two fitness functions were used to infer the network model from the dataset 1 described in the paper (i.e., the 25 nodes dataset). Table S1 depicts the result of the structural classification for using the original MSE function; table S2 is the result of adopting the fitness function with structural information. As shown in the two tables, the fitness function considering both the expression error and structural information outperforms the MSE function, in terms of the precision and recall rates. That is, 18.44% versus 51.38% for the precision rate, and 90.17% versus 97.11% for the recall rate, respectively.

Table S1. The structural classification matrix for dataset 1 (25 nodes) evaluated by the original fitness function.

|  |  | **Actual Class** | |  |
| --- | --- | --- | --- | --- |
|  |  | **Connection** | **No connection** |  |
| **Predicted Class** | **Connection** | 156  (true positive) | 690  (false negative) | Positive predictive rate (**precision**): 18.44% |
|  | **No** **connection** | 17  (false positive) | 387  (true negative) | Negative predictive rate: 95.79% |
|  |  | Sensitivity (**recall**): 90.17% | Specificity:  35.93% | Total Accuracy:  43.33% |

Table S2. The structural classification matrix for dataset 1 (25 nodes) evaluated by the proposed fitness function.

|  |  | **Actual Class** | |  |
| --- | --- | --- | --- | --- |
|  |  | **Connection** | **No connection** |  |
| **Predicted Class** | **Connection** | 168  (true positive) | 159  (false negative) | Positive predictive rate (**precision**): 51.38% |
|  | **No** **connection** | 5  (false positive) | 918  (true negative) | Negative predictive rate: 99.46% |
|  |  | Sensitivity (**recall**): 97.11% | Specificity:  85.24% | Total Accuracy:  86.88% |
